# The Deleterious Effects of Shiga Toxin Type 2 Are Neutralized In Vitro by FabF8:Stx2 Recombinant Monoclonal Antibody

**DOI:** 10.3390/toxins13110825

**Published:** 2021-11-22

**Authors:** Daniela Luz, Fernando D. Gómez, Raíssa L. Ferreira, Bruna S. Melo, Beatriz E. C. Guth, Wagner Quintilio, Ana Maria Moro, Agostina Presta, Flavia Sacerdoti, Cristina Ibarra, Gang Chen, Sachdev S. Sidhu, María Marta Amaral, Roxane M. F. Piazza

**Affiliations:** 1 Laboratório de Bacteriologia, Instituto Butantan, Sao Paulo 05503-900, Brazil; daniedaluz@gmail.com (D.L.); raissalozzardo@gmail.com (R.L.F.); brn.smelo@gmail.com (B.S.M.); 2 Laboratorio de Fisiopatogenia, Instituto de Fisiología y Biofísica Bernardo Houssay (IFIBIO Houssay-CONICET), Departamento de Fisiología, Facultad de Medicina, Universidad de Buenos Aires, Buenos Aires 1121, Argentina; gomezfernandoD@gmail.com (F.D.G.); agospresta@hotmail.com (A.P.); flasacerdoti@gmail.com (F.S.); cristinaadrianaibarra@gmail.com (C.I.); 3 Departamento de Microbiologia, Imunologia e Parasitologia, Universidade Federal de Sāo Paulo, Sao Paulo 04023-062, Brazil; bec.guth@unifesp.br; 4 Laboratório de Biofármacos, Instituto Butantan, Sao Paulo 05503-900, Brazil; wagner.quintilio@butantan.gov.br (W.Q.); ana.moro@butantan.gov.br (A.M.M.); 5 Banting and Best Department of Medical Research, Terrence Donnelly Centre for Cellular and Biomolecular Research, University of Toronto, Toronto, OT M5S 3E1, Canada; gchen2012@gmail.com (G.C.); sachdev.sidhu@utoronto.ca (S.S.S.)

**Keywords:** STEC, Stx2, antibody fragment, monoclonal antibody

## Abstract

Hemolytic Uremic Syndrome (HUS) associated with Shiga-toxigenic *Escherichia coli* (STEC) infections is the principal cause of acute renal injury in pediatric age groups. Shiga toxin type 2 (Stx2) has in vitro cytotoxic effects on kidney cells, including human glomerular endothelial (HGEC) and Vero cells. Neither a licensed vaccine nor effective therapy for HUS is available for humans. Recombinant antibodies against Stx2, produced in bacteria, appeared as the utmost tool to prevent HUS. Therefore, in this work, a recombinant FabF8:Stx2 was selected from a human Fab antibody library by phage display, characterized, and analyzed for its ability to neutralize the Stx activity from different STEC-Stx2 and Stx1/Stx2 producing strains in a gold standard Vero cell assay, and the Stx2 cytotoxic effects on primary cultures of HGEC. This recombinant Fab showed a dissociation constant of 13.8 nM and a half maximum effective concentration (EC_50_) of 160 ng/mL to Stx2. Additionally, FabF8:Stx2 neutralized, in different percentages, the cytotoxic effects of Stx2 and Stx1/2 from different STEC strains on Vero cells. Moreover, it significantly prevented the deleterious effects of Stx2 in a dose-dependent manner (up to 83%) in HGEC and protected this cell up to 90% from apoptosis and necrosis. Therefore, this novel and simple anti-Stx2 biomolecule will allow further investigation as a new therapeutic option that could improve STEC and HUS patient outcomes.

## 1. Introduction

The hemolytic uremic syndrome (HUS) in children is mostly caused by Shiga toxin-producing *Escherichia coli* (STEC) infection, which is also responsible for outbreaks in the United States, Europe, South America, and Japan [1,2,3]. In Argentina, where post-diarrheal HUS is endemic, around 300 new cases are reported each year [4]. Since the early 2000s, epidemiologically, the emergence of the non-O157 STEC infection, replacing the traditionally predominant O157 serogroup occurrence [5]. The contamination by STEC strains is usually by contaminated food or water ingestion, person-to-person transmission, or contact with ruminants or its contaminated environment [6]. The primary infection symptom is diarrhea, which is an average incubation phase of three days that could turn bloody in about 60% of patients. However, Shiga toxins (Stx) released by STEC triggers thrombogenic and inflammatory microvascular endothelial cell alterations, leading to HUS in 5–15% of STEC infection cases. HUS is defined by hemolytic anemia, thrombocytopenia, and acute renal injury [7,8]. Besides death, this syndrome can lead to long-term consequences such as hypertension and renal disease because of the high sensitivity to the Stx of the microvascular endothelial cells in the kidney [9].

The Stx toxins produced by STEC are Stx1 and Stx2, they appear to differ significantly in their effectiveness to induce protein synthesis inhibition and cytotoxicity, with some subtypes of Stx2 more potent than Stx1, on the other hand, other subtypes have similar potency [10]. Stxs is AB_5_ type toxin, consisting of a homo-pentameric B subunit (7.7 kDa per monomer) which binds to the host receptor globotriaosylceramide (Gb3) and mediate the enzymatically active A subunit (~32 kDa) endocytosis. Once inside the cell, the A subunit depurinates the conserved adenine residue of 28S eukaryotic rRNA, stopping peptide elongation and leading to cell death [11,12,13]. No specific drug has proved effective as specific therapy for STEC-HUS, which remains as symptomatic care. The antibiotics administration in STEC infection and STEC-HUS remains controversial, with some bacteriostatic antibiotics having a beneficial effect while others can increase the Stx liberation by the bacteria [14]. Proofs of evidence of an advantage from complement blockade therapy in STEC-HUS are also lacking [15]. One alternative treatment for STEC infection and possibly for HUS is neutralizing anti-Stx antibody therapy.

Monoclonal antibodies (mAb) against Stx have been evaluated in animal models (reviewed in [16,17]). Moreover, few mAbs candidates have also been tested in healthy volunteers during phase I studies [18,19]. In addition, a chimeric anti-Stx1 and Stx2 mAb was challenged in a phase II study in South America, but definite evidence of its therapeutic efficacy remains vague [20,21].

In addition to conventional antibodies, recombinant antibodies can be an attractive replacement to avoid animal immunization and other limitations of hybridoma technology, a successful, but cumbersome and costly approach to generate monoclonal antibodies [22,23]. In this context, we may include a family of Stx2B-binding VHHs that neutralize Stx2 in vitro at a nanomolar to the subnanomolar range [24] and the FabC11:Stx2 generated by phage display technology and produced very efficiently using bacterial protein synthesis systems which were able to prevent Stx2 toxicity to human kidney cells and in mice [25,26]. Therefore, the generation of such molecules and studies concerning their applicability will provide new therapeutic options for treating STEC infections to prevent or ameliorate HUS outcomes.

Herein, also employing phage display antibody library F [27], a monovalent FabF8:Stx2 was generated, and efficiently produced in the bacterial system with neutralizing qualities against Stx. We introduce a novel and simple antitoxin agent as a new therapeutic option for STEC infections therapy.

## 2. Results

### 2.1. Selection of FabF8:Stx2 from a Human Antibody Fragment Phage Display Library

**Figure 1:**
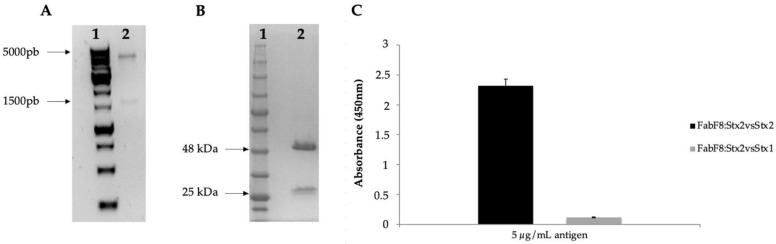
The FabF8:Stx2 generation. (**A**) FabF8:Stx2 gene cloning. Electrophoretic profile on 1.5% agarose gel stained with SYBR (1:1000) of restriction analyzes of FabF8:Stx2 clone. (1) 1Kb molecular weight marker (Invitrogen); (2) Clone F8 anti-Stx2 (FabF8:Stx2); (**B**) FabF8:Stx2 purification. Electrophoretic profile on 15% non-denaturing polyacrylamide gel stained with Coomassie blue of sample eluted from the purifications of Fab fragment. (1) Blueyed molecular weight marker (GE); (2) Clone F8 anti-Stx2. (**C**) ELISA assay to assess cross-reaction of ligands against Stx toxins (5 μg/mL) using EC_50_ concentration of FabF8:Stx2.

The FabF8:Stx2 was generated from the selection using purified Stx2a toxin and a human synthetic antibody phage display library (library F) developed by Persson et al. [27]. The cloning was confirmed by sequencing (Figure 1A). The 48 kDa fragment corresponds to the purified Fab fragment, however, a 25 kDa protein also appears, which corresponds to non-assembled variable chains (Figure 1B). As determined by surface plasmon resonance, the purified FabF8:Stx2 showed an affinity constant (K*_D_*) of 13.8 nM (Figure S1). The half-maximum effective concentration (EC_50_) was determined as being 160 ng/mL (calculated as described in the material and methods) as well as, specificity just for the selected toxin, with no significant cross-reactivity to Stx1 toxin (Figure 1C).

### 2.2. FabF8:Stx2 Neutralizes the Cytotoxic Effect of Supernatants from Different Stx2-Producing Strains

**Figure 2:**
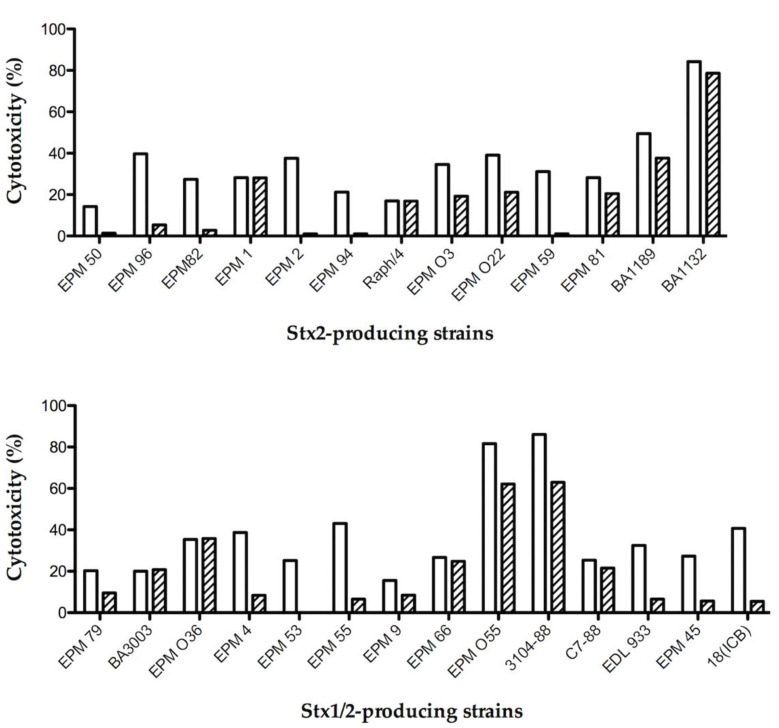
Percentage of cytotoxicity of STEC supernatants from strains bearing *stx2* or *stx1/2* genes; in absence (white bars) and in presence of FabF8:Stx2 (flared bars). Data represents biological duplicates of three independent experiments.

The FabF8:Stx2 was employed in a gold standard Vero cell assay (VCA) to test its neutralization ability to the toxicity of the supernatants from different STEC strains producing Stx2 or Stx1/2. The ability of this antibody in neutralizing the purified Stx2 was 84% (Table 1). Bacterial supernatant cytotoxicity was tested in the absence and presence of FabF8:Stx2 (Figure 2). It was observed that 85% of the tested supernatants were neutralized (from 7 to 100%), in 20 strains this rate was above 20% and this ability was superior to 40% in 16 strains. Therefore, the FabF8:Stx2 neutralizing ability ranged from 0 to 100%. No significant differences were observed in its neutralizing ability against Stx2 or Stx1/Stx2-producing strains. This recombinant antibody failed to neutralize only four strains, none of them producing just Stx2a (Figure 2, Table 1).

### 2.3. FabF8:Stx2 Protects Cell Viability of Human Glomerular Endothelial Cells (HGEC) from Stx2 Effects

**Figure 3:**
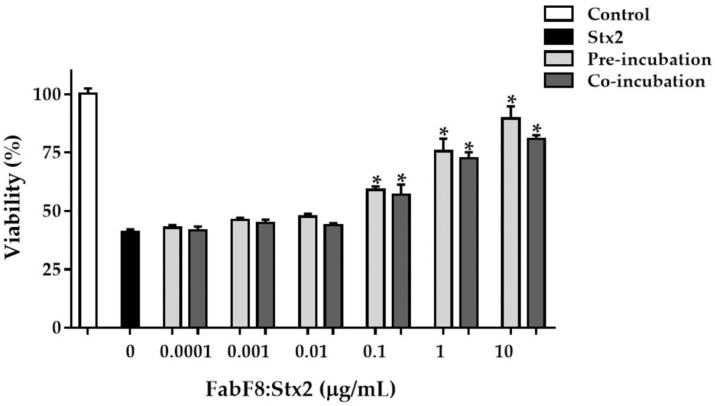
FabF8:Stx2 protects human renal endothelial cells (HGEC) against Stx2 cytotoxicity. HGEC were pre-treated with different concentrations of FabF8:Stx2 (1 h at 37 °C), and Stx2 (0.5 ng/mL) was then added, or cells were co-treated with FabF8:Stx2 (0.0001 µg/mL to 10 µg/mL) and Stx2 (0.5 ng/mL) simultaneously. Results are expressed as means ± SD of three experiments, pre/co-incubation vs. Stx2, * *p* < 0.05.

Considering the deleterious effects of Stx2 on the HGEC viability, we evaluate the FabF8:Stx2 ability to neutralize Stx2 cytotoxicity on HGEC. In a dose-dependent manner, the FabF8:Stx2 significantly neutralized the cytotoxic effects caused by 0.5 ng/mL Stx2 in HGEC (*p* < 0.05, *n* = 3) in both tested treatment conditions (pre-incubation or co-incubation). After the Stx2 treatment, the HGEC viability percentage was 41.0 ± 1.2%. The highest protection of HGEC viability was observed with 10 µg/mL of FabF8:Stx2 and no significant differences were found between pre-incubation and co-incubation, since cell viability percentage with 10 µg/mL FabF8:Stx2 was 89.6 ± 5.0% and 81 ± 1.7% for each condition, respectively (Figure 3). To calculate the percentage of FabF8:Stx2 protection, we first calculate the maximum prevention possible to obtain in the HGEC viability by subtracting the percentage of viability after Stx2 treatment to the viability of controls. Then, we calculated the additional % of HGEC viability obtained with FabF8:Stx2 by subtracting the viability % of Stx2 treated cells to pre-incubation and co-incubation. Finally, with these results, the percentage of protection with FabF8:Stx2 at both experimental conditions was calculated with the following formula: [(% of cell viability Pre/Co treated with FabF8:Stx2—% of cell viability in Stx2 treated cells)/(% of control cell viability—% of cell viability in Stx2 treated cells)] × 100. The protection obtained with 10 µg/mL FabF8:Stx2 was 83.0 ± 5.1% at the pre-incubation condition, and 67.5 ± 1.7% at the co-incubation condition, without statistical differences (Table 2).

### 2.4. FabF8:Stx2 Antibodies Prevent Detachment and Swelling Caused by Stx2 in HGEC

**Figure 4:**
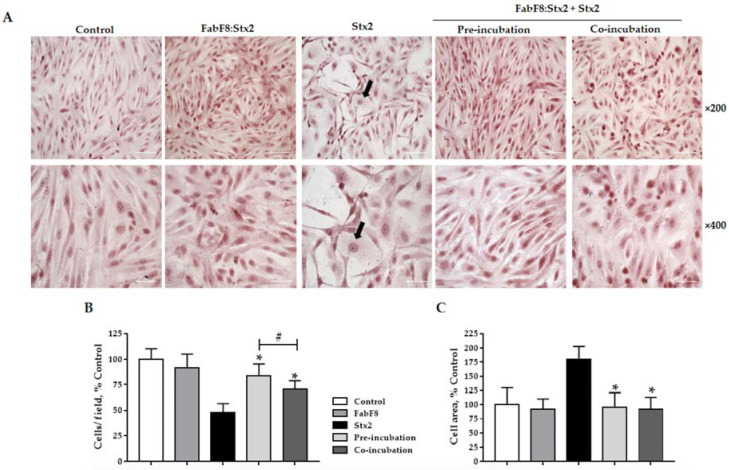
FabF8:Stx2 protects human glomerular endothelial cells (HGEC) from Stx2-induced morphological alterations (**A**) Cell morphology was evaluated and the number of HGEC (**B**) were analyzed by light microscopy (×200 and ×400). HGEC areas (**C**) were measured using Image J software. The black arrows indicate intracellular edema. Results are expressed as means ± SD of three experiments. One hundred percent represents the values of controls. Stx2 vs. Ctrl, * *p* < 0.05. Pre/co-incubation vs. Stx2, # *p* < 0.05.

Morphology of HGEC treated with Stx2 in the presence of FabF8:Stx2 antibodies was evaluated. This recombinant antibody fragment significantly prevented HGEC detachment and intracellular edema caused by 0.5 ng/mL Stx2 (Figure 4A). The prevention obtained on cell detachment with pre-incubation and co-incubation conditions was 62.0 ± 4.0 and 45.0 ± 3.0 (*p* < 0.05, *n* = 3), respectively. In addition, cell detachment protection was significantly greater with pre-incubation than co-incubation (Figure 4B). However, when the cell area was analyzed, practically a total protection from swelling was achieved with FabF8:Stx2 with both experimental conditions assayed, pre-incubation: 95.0 ± 6.0% and co-incubation: 90.0 ± 5.5%, *p* < 0.05, *n* = 3 (Figure 4C).

### 2.5. FabF8:Stx2 Antibodies Avoid Apoptosis Induced by Stx2 in HGEC

**Figure 5:**
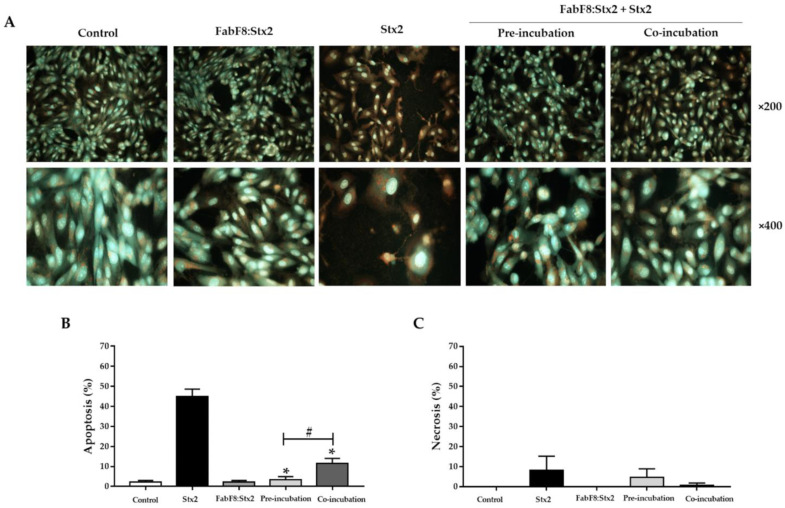
FabF8:Stx2 prevents apoptosis induced by Stx2 in human glomerular endothelial cells (HGEC). The percentage of necrotic and apoptotic cells after 72 h of treatments was established morphologically by fluorescence microscopy after staining with acridine orange/ethidium (×200 and ×400). A representative experiment is shown in panel (**A**). Results are expressed as means ± SD of three experiments. Apoptosis (**B**), * *p* < 0.05 for pre/co-incubation vs. Stx2. # *p* < 0.05 for pre-incubation vs. co-incubation. Necrosis (**C**), ns.

Apoptosis is the principal cell death mechanism triggered by Stx2. We previously showed that this toxin-induced necrosis and apoptosis in HGEC [28]. Following, we evaluated the ability of FabF8:Stx2 antibodies to avoid necrosis and apoptosis by analyzing HGEC stained with acridine orange/ethidium bromide by fluorescence microscopy (Figure 5A). The FabF8:Stx2 (1 µg/mL), significantly decreased the apoptosis caused by 0.5 ng/mL Stx2 in both experimental conditions (pre-incubation: 3.3 ± 0.9% and co-incubation: 11.6 ± 1.4 vs. Stx2: 45.0 ± 2.0%, *p* < 0.05, *n* = 3). Furthermore, FabF8:Stx2 at pre-incubation conditions was more effective than co-incubation to prevent apoptosis (93.0 ± 0.90 % vs. 75.0 ± 1.4%, respectively, *p* < 0.05, *n* = 3) (Figure 5B). No significant differences were found for necrosis (Figure 5C).

## 3. Discussion

Shiga toxin (Stx) is central to the development of hemolytic uremic syndrome (HUS). The supportive treatment is the current default procedure for STEC-infected patients, also, the administration of some antibiotic classes could increase the Stx production or release, which could lead to a risk of catastrophic consequences with HUS development, making this treatment option highly controversial [6,10]. Therefore, it is mandatory to develop either an effective treatment or a prevention method for the deleterious effects of Stx intoxication [19]. The STEC prevention is focused on individual and industry levels, such as hygiene procedures, meat processing protocols, and slaughterhouse maintenance, for example. Regarding therapy, it is focused mainly on inpatient supportive care, even though some strategies are in development aiming at different stages of infection, such as bacterial growth control without increasing Stx secretion, toxin trafficking interference and cellular response to the toxin. Moreover, it is worth mentioning the challenges regarding a therapeutic approach against Stx-induced symptoms, especially for clinical trials, such as the low incidence of STEC infections and HUS, the lack of highly specific diagnostic screening, and the narrow therapeutic window (onset of disease 3 days after infection, HUS development one week after the first symptom), which is also hypothetical [29]. Thus, despite great achievements towards a therapeutic tool against Stx, a specific treatment remains elusive.

Specific antibodies against Stx as a tool either to prevent or treat the HUS disease process is a promising approach [20]. Indeed, some other recombinant antibodies have also shown neutralizing ability against Stx2 in vitro or in vivo. Such as, the family Stx2B-binding VHHs which were constructed with one anti-Stx2B VHH, and two copies were fused to one anti-human albumin VHH, neutralizing Stx2 in vitro [24]. Another VHH also protected mice against Stx2 intoxication, but it was not humanized [30]. Concerning scFv, the one described by Maa et al. [31] and Luz et al. [25], neutralizes the cytotoxic ability of Stx2 in vivo and in vitro, respectively, however, none of them is a human antibody, produced in a bacterial system, which impairs their use as therapeutic agents and costs of production.

The recombinant human Fab and F(ab’)2 fragments characterized by Akiyoshi et al. [32] showed neutralizing capacity, however, the production was dependable on mammalian cells (CHO), having a high cost for obtaining as with hybridoma technology. Therefore, the library F [27] was employed to select specific Fab high binders against Stx toxins. Two phage clones showed high affinity and binding ability against Stx2. The FabC11:Stx2 was the first to be characterized and showed cross-reactivity with Stx1 besides being able to prevent Stx2 toxicity to human kidney cells and in mice [21,22].

Herein, the other Fab selected against Stx2 (FabF8:Stx2) was characterized and employed in different cell assays. The variety of toxin subtypes that could be expressed by a diverse set of STEC serotypes able to express one or more toxin types at the same time is a major challenge for antibody-based Shiga toxin neutralizers, once to be universally effective, should neutralize multiple Stx1 and Stx2 subtypes [14]. In the present study, using the gold standard Vero cell neutralization assay, we observed that FabF8:Stx2 neutralized the cytotoxicity of 23 of 27 supernatants from Stx2 or Stx1/Stx2-producing STEC strains. These strains belong to different serotypes and present diverse *stx* subtypes, it is worth mentioning that no differences were found with neutralization percentage and its *stx* subtype, even though the FabF8:Stx2 was generated against a Stx2a toxin, some strains harboring *stx2a* gene were not neutralized whereas two non-Stx2a producing were neutralized. This kind of investigation is not commonly employed, usually, most neutralization assays are tested against one type and/or one subtype of the toxin, therefore in this work, for the first time, we showed how one recombinant monoclonal antibody neutralizes different Stx combinations obtained from the STEC isolates culture.

The human microvascular endothelial cells are an excellent cell model for in vitro therapeutic studies once it can express 50-fold higher Gb_3_ levels compared to the endothelial cells of large vessels [33]. In this sense, previously, we developed human glomerular endothelial cells (HGEC) primary cultures and demonstrated the decrease of cell viability by apoptosis and endothelial injury like that documented in kidney biopsies of HUS patients after incubation with Stx2 [28]. In this work, additionally, we assayed FabF8:Stx2 antibodies on HGEC exposed to Stx2 and we were able to corroborate their great effectiveness on the protection of Stx2 cytotoxicity on HGEC, in about 80–90% at the pre-incubation condition. These results were coincident with the high capacity of these antibodies to prevent HGEC apoptosis in about 75–90% under pre-incubation and co-incubation conditions. Furthermore, at pre-incubation, cell detachment was avoided in approximately 60–65% and swelling, in about 90–95%.

Previously, we demonstrated that by using 10 µg/mL FabC11:Stx1/Stx2 we observed lower protection of the HGEC viability (54.0% and 52.0%, for pre-incubation and co-incubation, respectively) compared to the same concentration of FabF8:Stx2 when cells were exposed to a 1 CD_50_ of Stx2, therefore, preventing Stx2 toxicity on human kidney cells [26]. Additionally, FabC11:Stx1/Stx2 cell detachment protection was also lower than FabF8:Stx2, which showed protection levels of 43.5% under pre-incubation and 36% under co-incubation conditions. With respect to swelling, although we demonstrated good prevention, pre-incubation: 97.0% and co-incubation: 81.0%, it is noteworthy that this protection had been obtained with a higher concentration of FabC11:Stx1/Stx2 (10 µg/mL) compared with FabF8:Stx2 (1 µg/mL). Our results conclusively demonstrate the efficacy of FabF8:Stx2 antibodies to avoid the cytotoxic effects of Stx2 on human renal microvascular endothelial cells, one of the principal target cells for this toxin, raising the possibility of the development of a new therapeutic molecule against Stx2 toxicity.

## 4. Conclusions

The present work showed encouraging results about the effectiveness of FabF8:Stx2 antibodies to neutralize the cytotoxic effects of both purified Stx2 and Stx2 or Stx1/Stx2 produced by STEC strains. Thus, they could be a promising therapeutic strategy to prevent kidney damage and the subsequent development of HUS. Future studies will be focused on analyzing the efficacy of FabF8:Stx2 in in vivo models.

## 5. Materials and Methods

### 5.1. Bacterial Strains, Plasmids, and Antigen

The bacterial Phage-resistant *Escherichia coli* Omnimax (Invitrogen) was used for Phage Display assays. For Fab cloning and expression, *Escherichia coli* DH5a (Thermo Fisher Scientific) and BL21(DE3) pLysS (Novagen) were used, respectively. The plasmid vectors used were phagemid HP153 and pFabHis-MBP [25]. The bacterial strains used in this study were strains previously defined as STEC by gene presence and Stx1 or Stx2 production [34], including the prototype EDL933 (O157:H7) [35] (Table 1). The antigens Stx2 and Stx1 were commercially available and acquired from Phoenix Laboratory, Tufts Medical Center, Boston, MA, USA.

### 5.2. Antibody Generation and Characterization

The FabF8:Stx2 was selected by phage display, using a human synthetic antibody library (library F), which displays on the M13 bacteriophage surface a Fab antibody fragment [27]. Selection and panning were performed as described by Sidhu and Fellouse [36] using the protocol of selection against immobilized antigens. In summary, it was used 5 μg/mL toxin (100 μL/well) in phosphate-buffered saline (PBS) to coat a microplate (Maxisorp, Nunc) in the first round and 2.5 μg/mL toxin (100 μL/well) in PBS for the followed selection rounds. The same was performed with the negative protein control (MPB). The coated plate was incubated at room temperature for 2 h or 18 h at 4 °C, followed by blocking for 1 h with 200 μL/well PBS-0.2% BSA. Next, the phage library solution in PBT buffer (PBS-0.2% BSA-0.05% Tween-20) was added to the negative control wells (100 μL/well) and the plate was incubated at room temperature for 2 h with gentle shaking. The content of the control wells containing the phage library solution was removed and placed into the toxin-coated wells. Next, the non-binded phages were removed by washing them 10 times with PT buffer (PBS-0.05% Tween-20). Toxin-bound phages were eluted by adding 100 μL/well of 100 mM HCl and incubating at room temperature for 5 min. To neutralize the pH of the eluent, it was transferred to a new 1.5-mL microfuge tube containing 1.0 M Tris-HCl, pH 11. Half of the eluted phage solution was added to 10 volumes of actively growing *E. coli* omnimax (OD600 < 1.0) in 2YT/tet medium, which was then incubated at 37 °C for 20 min with shaking at 200 rpm before M13-K07 helper phage were added (10^10^ infectious units (IU)/mL) and the whole culture was incubated at 37 °C with shaking for an additional hour. The culture was then transferred to a 30 mL 2YT/carb/kan medium, and cells grew at 37 °C overnight before phage was harvested for the next round of panning. A serial dilution on LB/carb plates was performed to determine the number of phages eluted and four panning rounds were performed. The phage selected was sequenced and forwarded to cloning and production. The cloning of the Fab expression vector, Fab fragment expression, and purification was performed as previously reported by Luz et al. [25].

#### FabF8:Stx2 Characterization

*Surface Plasmon Resonance*—The antibody affinity was determined by surface plasmon resonance (BIAcore T200, Cytiva, Little Chalfont, UK) following the manufacturer’s recommendations. The experiments used HBS-EP buffer, pH 7.4, containing 10 mM HEPES, 150 mM NaCl, 3 mM EDTA, and 0.05% Tween 20 as the running buffer. Briefly, Stx2 (purchased from Tufts University School of Medicine, Boston, MA, USA) at 5 µg/mL in 10 mM sodium acetate buffer, pH 5.5 was immobilized (152 RU) on CM5 sensor chips activated by mixing equal amounts of *N*-ethyl-*N*’-(dimethyl aminopropyl) carbodiimide (EDC) and *N*-hydroxysuccinimide (NHS) following the standard immobilization protocol. The sample preparation was in HBS-EP buffer (0–7.4 µM, twofold dilutions) and the kinetic study was performed by a multicycle model at 25 °C and a flow rate of 30 µL/min (contact of 120 s and dissociation of 600 s). The sensor chip was regenerated between cycles by a 15 µL pulse of 100 mM glycine containing 2 mM MgCl_2_, pH 2. The kinetic affinity constant (KD) was calculated using BIAevaluation version 3.0, using the Langmuir 1:1 binding model. Stx2 monoclonal antibody was employed as a control [36]. The experiments were performed in duplicate.

*EC_*50*_ definition*—Half-maximal effective concentration (EC_50_) was performed as described by Luz et al. [25] by coating a 384-well plate (Maxisorp) with 2 μg/well of antigen and incubating overnight at 4 °C with gentle shaking, followed by blocking step with 0.2% PB buffer for 1 h at room temperature with gentle shaking. A log 3 serial Fab/scFv dilutions, starting with 20 μg/mL, were performed in PBT, and incubated for 30 min at room temperature with gentle shaking. The assay development was performed after 30 min incubation with gentle shaking using HRP antibody/anti-Flag conjugated to peroxidase (1:5000) in PBT followed by addition of TMB (1:1) and stop with 1 M H_3_PO_4_. Several washes with PBT were performed between each incubation. The plate was read with a 450 nm filter. Specificity and absence of cross-reactivity were performed by ELISA as described by Luz et al. [25] using a 96-well plate (Maxisorp) coated with different concentration Stx2 or Stx1 purified toxins (5 μg/mL) incubated 18 h at 4 °C with gentle shaking, followed by blocking with 0.2% PB buffer for 1 h at room temperature with gentle shaking. The EC_50_ concentration of FabF8:Stx2 was added to the plate and incubated for 30 min at room temperature with gentle shaking, followed by 8 times washing with PT. Next, it was added (100 μL/well) into the wells, HRP antibody/anti-Flag conjugated to peroxidase (1:5000) in PBT, which was then incubated for 30 min at room temperature with gentle shaking. Again, the plate was washed 8 times with PT. The reaction was developed by adding 30 μL/well of TMB (1:1) and stopped by adding 30 μL/well of 1 M H_3_PO_4_, and the plate was read with a 450 nm filter.

### 5.3. Vero Cell Antibody Neutralization Assay

The certified Vero cell lineage was purchased from Instituto Adolfo Lutz (São Paulo, SP, Brazil). The STEC bacterial supernatant was obtained as described by Shiga et al. [37]. Vero cells (1 × 10^5^ cells/mL) were grown in 96-well plates in Dulbecco’s medium (DMEM) supplemented with 10% FBS and 30 μg/mL gentamicin, at 37 °C in a 5% CO_2_ atmosphere, for 24 h. The FabF8:Stx2 neutralizing ability was determined by pre incubating for 2 h the Stx2 toxin at the CD_50_, (i.e., 0.5 μg/mL) defined by Rocha et al. [38] or bacterial supernatants (diluted 1:50) with the same volume of an EC_50_ concentration of Fab diluted in DMEM supplemented with 2% of FBS at 37 °C for 72 h with 5% CO_2_. After incubation, the viable cells were accessed by 3-(4,5-dimethylthiazol-2-yl)-2,5-diphenyltetrazolium bromide (MTT) (Sigma-Aldrich, St Louis, MO, USA) as described by the manufacturer’s instructions. These assays were performed three times in duplicate.

### 5.4. Primary Culture

The human glomerular endothelial cells (HGEC) were obtained as previously described by Amaral et al. [28] from kidneys of human pediatric patients, under proper consent and ethical approval (N°: 035 LUP1S0/19). HGEC were cultivated in M199 media, supplemented with 20% fetal calf serum (FCS), 3.2 mM L-glutamine, 100 U/mL penicillin/streptomycin (GIBCO, Waltham, MA, USA), and 25 µg/mL endothelial cell growth supplement (ECGS, Sigma, St. Louis, MO, USA). All the experiments were performed with HGEC between 2–7 passages and were previously characterized for positive expression of von Willebrand factor and platelet/endothelial cell adhesion molecule 1 (PECAM-1). Moreover, experiments were carried out at growth-arrested conditions using M199 medium supplemented with 10% FCS without ECGS [28].

### 5.5. Stx2 Neutralization Assay in HGEC

Neutralization assays were developed according to two procedures: pre-incubation and co-incubation. The pre-incubation was performed by pre-treat HGEC primary cultures with different FabF8:Stx2 concentrations (1 h at 37 °C) before exposure of cells to Stx2 for 72 h. On the other hand, for co-incubation, cells were treated simultaneously with FabF8:Stx2 and Stx2 for 72 h. FabF8:Stx2 concentrations used in the experiments ranged from 0.0001 to 10 µg/mL and Stx2 was assayed at the dilution required to kill 50% of cells (1 CD_50_ = 0.5 ng/mL). The molar ratios used ranged from 0.07:5 to 7000:5 for FabF8:Stx2. Finally, the ability of FabF8:Stx2 to neutralize Stx2 was analyzed by HGEC cell viability as is described below.

### 5.6. Neutral Red Viability Assay

The neutral red uptake assay was used to analyze the HGEC cell viability as previously described [28]. In summary, HGEC cells were grown with a complete medium, until confluence, in 96-well plates. After 72 h of treatments, freshly diluted neutral red (Sigma Aldrich, St. Louis, MO, USA) was added to cells to a final concentration of 10 mg/mL, followed by an additional incubation of 1 h at 37 °C in 5% CO_2_. Then, cells were washed and fixed with 1% CaCl_2_/1% formaldehyde, followed by lysis with 1% acetic acid in 50% ethanol. Absorbance at 540 nm was measured in an automated plate spectrophotometer. Results were expressed as viability percentage, in which 100% represents cells incubated under identical conditions but without treatment. The 100% of HGEC viability protection was considered when Stx2 cytotoxic effects were totally reversed.

### 5.7. Cell Morphology Analysis

HGEC cells were grown on gelatinized glass coverslips (12 mm) and treated as it was described above. For cell morphology analysis, FabF8:Stx2 were used at 1 µg/mL. Following, cells were fixed with 96% v/v alcohol for 2 h at room temperature and stained with hematoxylin/eosin (H&E). Subsequently, HGEC were analyzed by light microscopy (×200 and ×400, Zeiss Axiophot, Zeiss, Heidelberg, Germany). The percentage of cells/field was obtained from photographs of 10 randomly selected fields. Cells were then counted and averaged, and the percentage of cells per field was estimated by considering the average number of controls as 100% (percentage of cells/field = (number of treated cells × 100)/number of control cells). Furthermore, the percentage of cell area was calculated from the same photographs. For that, the cell area was analyzed in each cell by using the Image J software (NIH) according to the manual instructions. The cell area average was calculated for each condition and the cell area of controls was considered as 100% (percentage of cell area/field = (cell area of treated cells × 100)/cell area of control cells [28]. Results were expressed as means ± standard deviation of the mean (SD). The percentages of protection from cell detachment and intracellular edema were calculated considering 100% prevention when these alterations were totally reversed.

### 5.8. Necrosis and Apoptosis Analysis

HGEC were grown on gelatinized glass coverslips (12 mm) and then treated as it was described for item 5.7. After treatments, the percentage of necrotic and apoptotic cells were analyzed morphologically by fluorescence microscopy. For that, cells were stained with acridine orange/ethidium bromide (1:1, v/v) at a final concentration of 100 μg/mL [28]. In the analysis, it was considered that live cells have normal nuclei when presented with green chromatin and organized structures. Apoptotic cells contain fragmented or condensed chromatin (green or orange). Finally, necrotic cells have similar normal nuclei staining as live cells, but with the chromatin in orange instead of green. The percentage of apoptotic and necrotic cells/field was obtained from photographs of 10 randomly selected fields. Cells were then counted, and the percentage of necrotic and apoptotic cells was estimated by considering the total number of cells/field as 100% (percentage of necrotic or apoptotic cells/field = (number of necrotic or apoptotic cells × 100)/total number of cells). Results were expressed as means ± standard deviation of the mean (SD). Percentages of prevention from necrosis and apoptosis were calculated by considering 100% of protection when these alterations were totally reversed.

### 5.9. Data Analysis

The data are presented as mean ± SD. ANOVA was used to calculate differences between groups and Tukey’s multiple comparisons test was used as a posteriori. All Statistical analysis was performed using Graph Pad Prism Software 5.0 (San Diego, CA, USA).

## Figures and Tables

**Table 1:** Stx-producing strains features.

Strain	Serotype	Source	Stx	Subtype	Neutralization Rate (%)
EPM 50	O87:H16	Animal	Stx2	2b	90
EPM 96	O93:H19	Food	Stx2	2a, 2d	86
EPM 82	O112:H21	Animal	Stx2	2c	90
EPM 1	O157:H7	Human	Stx2	2a, 2c	0
EPM 2	O157:H7	Human	Stx2	2a, 2c	97
EPM 94	O157:H7	Animal	Stx2	2c	95
Raph/4	O165:H^-^	Human	Stx2	2a, 2c	0
EPM O3	O172:NM	Animal	Stx2	2a	44
EPM O22	ONT:H16	Animal	Stx2	2b	46
EPM 59	ONT:H16	Animal	Stx2	2d	97
EPM 81	ONT:H38	Animal	Stx2	2a	27
BA 1189	ONT:H49	Human	Stx2	2a, 2d	24
BA 1132	ONT:H49	Human	Stx2	2a, 2c, 2d	7
EPM 79	O22:H16	Animal	Stx1/2	1a, 2c, 2d	53
BA 3003	O48:H7	Human	Stx1/2	1a, 2a	0
EPM O36	O75:H8	Animal	Stx1/2	1c, 2b	0
EPM 4	O93:H19	Human	Stx1/2	1a, 2d	78
EPM 53	O98:H17	Animal	Stx1/2	1a, 2a, 2c	100
EPM 55	O98:H17	Animal	Stx1/2	1a, 2a, 2c	85
EPM 9	O103:H2	Human	Stx1/2	1a, 2c	46
EPM 66	O105:H18	Animal	Stx1/2	1a, 2a, 2b	7
EPM O55	O146:H21	Animal	Stx1/2	1a, 2a, 2b	24
3104-88	O157:H7	Human	Stx1/2	1a, 2a	27
C7-88	O157:H7	Human	Stx1/2	1a, 2NT	15
EDL 933	O157:H7	Food	Stx1/2	1a, 2a	80
EPM 45	O181:H4	Animal	Stx1/2	1a, 2a	80
18 (ICB)	ND	ND	Stx1/2	1NT, 2NT	87
Purified Stx2	-	-	Stx2	2a	84

ND—not determined; NT—not typeable.

**Table 2:** Percentage of FabF8:Stx2 protection against Stx2 in HGEC cells.

FabF8:Stx2 (g/mL)	Stx2 Cytotoxicity Prevention (%)	
	Pre-Incubation	Co-Incubation
0	0	0
0.1	30.5 ± 1.5	27.1 ± 4.2
1	58.5 ± 5.4	53.5 ± 2.6
10	83.0 ± 5.1	67.5 ± 1.7

## Data Availability

The data presented in this study are available on request from the corresponding authors. The data are not available in the repository of the Butantan Institute (https://repositorio.butantan.gov.br (accessed on 16 November 2021).

## Supplementary Materials

The following are available online at https://www.mdpi.com/article/10.3390/toxins13110825/s1, Figure S1: Binding of Fab:F8 to Stx2 measured by Biacore.
