# Supplementary material for: The Deleterious Effects of Shiga Toxin Type 2 Are Neutralized In Vitro by FabF8:Stx2 Recombinant Monoclonal Antibody
Source: Toxins (Basel). 2021 Nov 22;13(11):825. doi: 10.3390/toxins13110825 (PMC8621789; doi:10.3390/toxins13110825)
Supplement: Supplementary file 1 [file toxins-13-00825-s001.zip › toxins-1442708-supplementary.pdf]

Supplementary Materials: The Deleterious Effects of Shiga Toxin Type 2 Are Neutralized In Vitro by FabF8:Stx2 Recombinant Monoclonal Antibody

Daniela Luz, Fernando D Gómez, Raíssa L Ferreira, Bruna S Melo, Beatriz EC Guth, Wagner Quintilio, Ana Maria Moro, Agostina Presta, Flavia Sacerdoti, Cristina Ibarra, Gang Chen, Sachdev S Sidhu, María Marta Amaral and Roxane MF Piazza

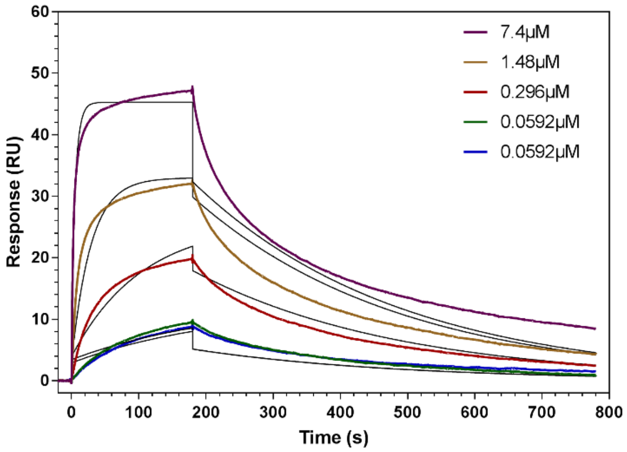

Figure S1. Binding of Fab:F8 to Stx2 measured by Biacore. Stx2 was immobilized on a CM5 sensorchip. The analytes consisted of serial dilutions of Fab F8 between 0 and 7.4 μM. Data was fitted in a Langmuir 1:1 interation model, using Biacore Evaluation software. Color lines: observed curves; Black lines: fitted curves.
